# Application of machine learning for multi-community COVID-19 outbreak predictions with wastewater surveillance

**DOI:** 10.1371/journal.pone.0277154

**Published:** 2022-11-10

**Authors:** Yuehan Ai, Fan He, Emma Lancaster, Jiyoung Lee

**Affiliations:** 1 Department of Food Science and Technology, The Ohio State University, Columbus, OH, United States of America; 2 Division of Environmental Health Sciences, College of Public Health, The Ohio State University, Columbus, OH, United States of America; 3 Environmental Science Graduate Program, The Ohio State University, Columbus, OH, United States of America; 4 Infectious Diseases Institute, The Ohio State University, Columbus, OH, United States of America; University of California San Francisco, UNITED STATES

## Abstract

The potential of wastewater-based epidemiology (WBE) as a surveillance and early warning tool for the COVID-19 outbreak has been demonstrated. For areas with limited testing capacity, wastewater surveillance can provide information on the disease dynamic at a community level. A predictive model is a key to generating quantitative estimates of the infected population. Modeling longitudinal wastewater data can be challenging as biomarkers in wastewater are susceptible to variations caused by multiple factors associated with the wastewater matrix and the sewersheds characteristics. As WBE is an emerging trend, the model should be able to address the uncertainties of wastewater from different sewersheds. We proposed exploiting machine learning and deep learning techniques, which are supported by the growing WBE data. In this article, we reviewed the existing predictive models, among which the emerging machine learning/deep learning models showed great potential. However, most models are built for individual sewersheds with few features extracted from the wastewater. To fulfill the research gap, we compared different time-series and non-time-series models for their short-term predictive performance of COVID-19 cases in 9 diverse sewersheds. The time-series models, long short-term memory (LSTM) and Prophet, outcompeted the non-time-series models. Besides viral (SARS-CoV-2) loads and location identity, domain-specific features like biochemical parameters of wastewater, geographical parameters of the sewersheds, and some socioeconomic parameters of the communities can contribute to the models. With proper feature engineering and hyperparameter tuning, we believe machine learning models like LSTM can be a feasible solution for the COVID-19 trend prediction via WBE. Overall, this is a proof-of-concept study on the application of machine learning in COVID-19 WBE. Future studies are needed to deploy and maintain the model in more real-world applications.

## Introduction

Wastewater-based epidemiology (WBE) has been employed as a complementary tool for COVID-19 monitoring worldwide since the beginning of the pandemic [1, 2]. One of the most valued benefits provided by WBE is its capability to give an early signal for the changing trend of a pandemic at a community level [3–5]. Timely actions can be taken when an onset trend is observed.

A recent study summarized that reasonable lead time for WBE and clinical report range from 0–4 and 0–6 days, respectively, depending on various factors, such as the clinical testing lag, result reporting delay, WBE result turnaround time, and the viral shedding dynamic in the stool [6]. Another study in Greece reported tan hat increase in the RNA load in wastewater lean to the increase in positive COVID-19 cases and hospitalization by 5 and 8 days [7]. When clinical testing capability is limited, the lead time of WBE tends to increase [8]. Meanwhile, as wastewater surveillance covers many locations across the United States and worldwide, WBE can provide near real-time data [9]. In this case, it can be expected that the lead time of WBE results will subsequently increase. Given this, wastewater COVID-19 surveillance will gain importance in this prolonged pandemic.

Another advantage of wastewater surveillance is that community-level data is collected instead of individual testing data, which enhances data privacy. Moreover, signals from both pre-symptomatic and asymptomatic carriers can be captured in wastewater [10]. Globally, wastewater service covers approximately 2.1 billion people who could benefit from health information provided by WBE [11]. For areas with limited clinical resources, WBE can be a cost-effective means of community-level monitoring to reduce the burden of massive individual tests [4].

However, few challenges exist with tracking COVID-19 via wastewater surveillance. A major criticism of WBE is associated with the uncertainties of making quantitative predictions on infected cases from the viral genetic marker concentration in wastewater [12]. For COVID-19 case prediction, the inaccuracy can result from the fluctuation of SARS-CoV-2 RNA concentration due to the variations in environmental and biochemical attributes of the wastewater matrix. The RNA of nonintact SARS-CoV-2 viral particles is susceptible to degradation by RNases. A variety of wastewater attributes can lead to the lysis of the virus, including but not limited to the travel time of the virus in the sewer system, wastewater temperature, and pH [13]. Moreover, the difficulties in standardizing the wastewater sampling and viral concentrating techniques can also lead to inaccurate case count estimation. Different models have been developed to tackle these uncertainties more accurately predict COVID-19 dynamics from wastewater. This modeling is crucial for scaling up wastewater surveillance as compensation for less individual testing in the future.

Conventional statistical models for predicting COVID-19 infection cases from wastewater were proposed in the early stage of the global pandemic. Most of them are regression-based epidemiological models with very few wastewater parameters included. More recent studies implemented various models for case prediction and forecasting from wastewater, including but not limited to the susceptible-exposed-infectious-recovered (SEIR) model, vector autoregression, and machine learning/deep learning models (Table 1). Among all the models, computational modeling methods, mainly machine learning and deep learning, showed great predictive potency.

**Table 1:** Summary of WBE models for COVID-19 surveillance.

Study Location	Predictive Model Type	Model Input	Model Output	Model Performance	Reference
Spain (1 sewershed); USA (6 sewersheds); USA (9 sewersheds)	Linear regression, Generalized additive model; Linear regression; Polynomial regression	SARS-CoV-2 viral load (N gene), Mean flowrate, Fecal indicator concentration	Active COVID-19 cases	R^2^ = 0.894; Root mean standard error of 13.1 cases per 100,000 population for the entire model; R^2^ ranged from 0.47–0.84	[14–16]
Australia (2 sewersheds)	Monte Carlo simulation	SARS-CoV-2 viral load (N gene), Flowrate, Per capita wastewater rate, SARS-CoV-2 shedding rate	Total infected individuals	Median SARS-CoV-2 infection prevalence agreed with clinical observations	[3]
United States (3 sewersheds)	Susceptible-exposed-infectious-recovered (SEIR) model	SARS-CoV-2 viral load (N gene), SARS-CoV-2 epidemiological metrics	Total infected individuals (unreported cases are 11 times that of confirmed cases)	Infections for each confirmed case statistically similar to other studies.	[17, 18]
Greece (1 sewersheds)	Linear regression (short-term prediction), Artificial neural networks (long-term prediction)	SARS-CoV-2 viral load (N gene), Confirmed COVID-19 cases	Admission cases, ICUs cases	Admission cases: Short term-R^2^ = 0.888, Long term-R^2^ = 0.924	[7]
Greek (2 sewersheds)	Linear regression, Random forest	SARS-CoV-2 viral load (ORF1ab, the Spike ORF and the Nucleocapsid ORF), Wastewater biochemical parameters (pH, electrical conductivity, total suspended solids, total nitrogen, etc.)	7-day cumulative COVID-19 active cases	Mean relative errors ranged from 30.42% to 59.46%	[19]
United States (1 sewershed)	Vector autoregression model	SARS-CoV-2 viral load (N gene),	1–3 weeks forecasting of active COVID-19 cases	Mean Absolute Percentage Error for 1-week forecasting: 11.85%	[20]
Canada (2 sewersheds)	Convolutional neural networks	SARS-CoV-2 viral load (N gene), Fecal indicator concentration, flowrate, biochemical oxygen demand, chemical oxygen demand	1–7 days forecasting of active COVID-19 cases	Wastewater data increased 5–7 day forecast accuracy by 42% than epidemiological data only	[21]

Since the beginning of the COVID-19 pandemic, machine learning techniques have been applied in contact tracing, outbreak forecasting, diagnosis, and recommending control strategies [12]. Outbreak forecasting was mainly performed at the national or global level. With WBE data, prediction or forecasting can be conducted to serve small communities. Though the implementation of machine learning in WBE is limited, it is a promising research direction as machine learning can provide a solution to the challenges of COVID-19 wastewater surveillance. First, wastewater surveillance data from diverse communities accumulates worldwide, yielding massive longitudinal data to explore. Second, machine learning can help resolve the uncertainties induced by the wastewater matrix. The dilution nature of wastewater biomarkers can pose significant challenges to the estimation of the infected population. With more biochemical and geographical features extracted from wastewater and its associated sewersheds, machine learning, especially deep learning models, can assess the temporal fluctuation of biomarkers. Some machine learning techniques hold intrinsic merit in handling multidimensional data, which aligns well with wastewater data. Here we suggest machine learning as an effective tool for COVID-19 trend prediction and forecasting. In this study, the main goal of applying WBE in small sewersheds/communities is to get an accurate prediction of the current and in-coming COVID-19 status. Therefore, the short-term predictive model was focused instead of long-term future forecasting. This is critical for host spot identification when the clinical testing is backlogged and insufficient.

Existing machine learning or deep learning models in COVID-19 WBE research have two major limitations. They are non-time series models and hardly mine any time-related features. In this long-term pandemic, time is an intrinsic and important factor that can impact the evolution of the outbreak. Seasonal trends and holiday surges have been widely observed [22]. Moreover, sewersheds are analyzed individually. As more and more wastewater facilities, agencies and laboratories get involved in WBE, machine learning models that support multi-site monitoring will benefit larger-scale surveillance and networking among the research facilities and government agents. This kind of model can provide data with less regional bias, which is critical for decision-making and allocating medical and economic resources.

**Fig 1:**
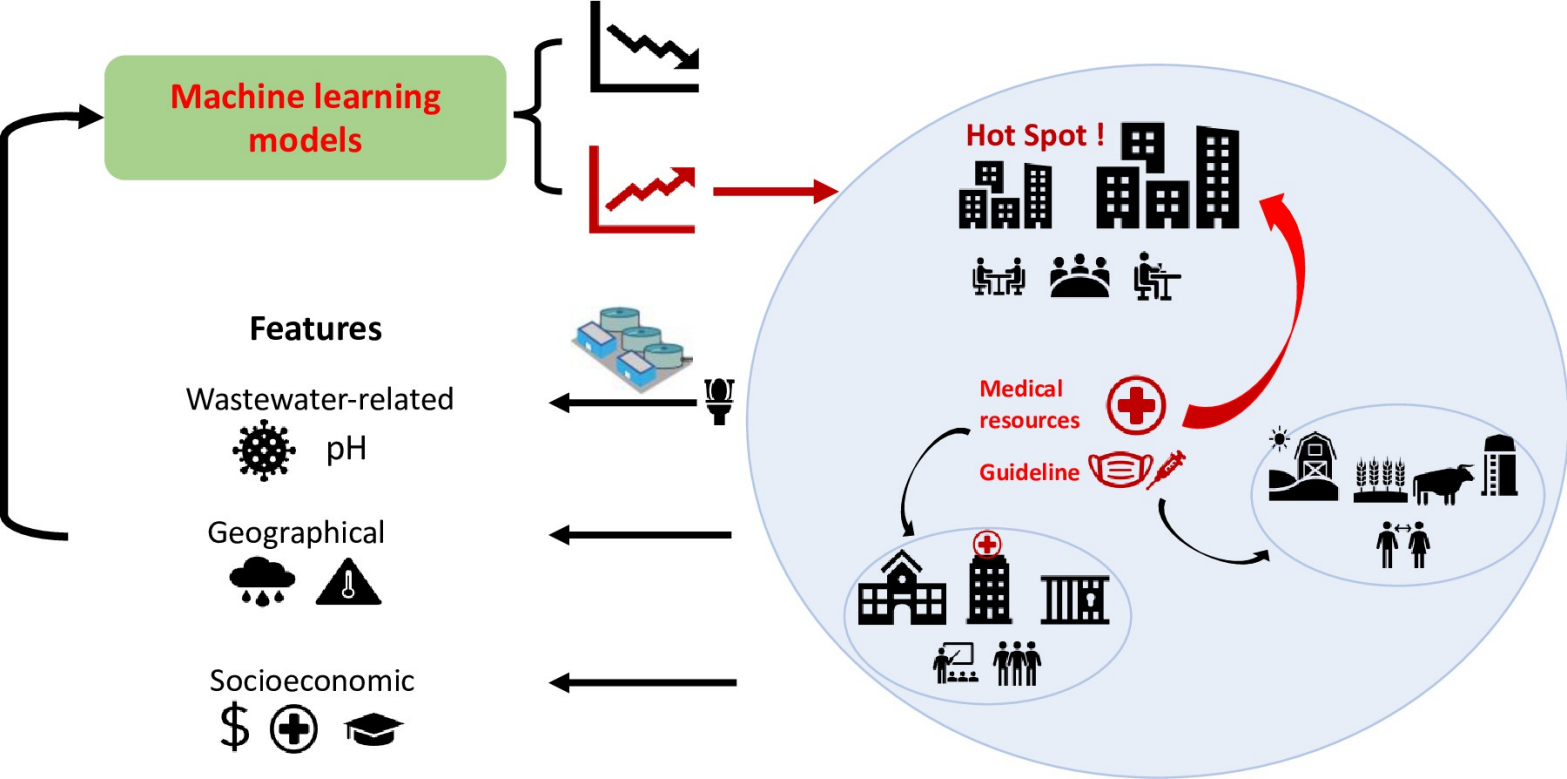
Concept of applying machine learning for multi-community COVID-19 outbreak predictions with wastewater surveillance.

In this study, we propose applying predictive time-series machine learning and deep learning models to track COVID-19 outbreaks in multiple communities (Fig 1). Thus, proof-of-concept modeling was conducted with three main objectives: (1) comparing the performance of different time series and non-time series machine learning models; (2) extracting different types of wastewater- and sewershed-related features and investigating their effects on the models; and (3) examining the potential lead time of wastewater surveillance to clinical reports.

## Materials and methods

### Predictive models

Five supervised machine learning models were implemented in this study to predict COVID-19 cases from the community in a sewershed using SARS-COV-2 viral loads in wastewater and other sewershed/community-related features. Firstly, three widely used non-time series machine learning models were developed, including multiple/univariate linear regression (MLR/LR), gradient boosting decision tree (GBDT), and feed-forward deep neural network (DNN) [23, 24]. As COVID-19 case data can contain temporal information, two more popular time-series models were also examined: Facebook Prophet and long short-term memory (LSTM). Prophet is an open-source general additive model for time series data [25]. LSTM is an advanced, recurrent neural network for long sequential data, which can also address the gradient explosion/vanishing problem [26]. It is not expected for the COVID-19 case series to be stationary, so time-series models like autoregressive integrated moving average (ARIMA) are not considered. LSTM and Prophet are known to be good at handling the seasonality and trends in the data.

### Wastewater samples and sewersheds

Six hundred twenty wastewater samples were obtained twice a week from nine sewersheds in central Ohio in the United States between September 2020 and June 2021. The sewersheds represent a diversity of communities in urban and rural areas, serving a population ranging from 14,000 to 900,000. More details about the sewersheds were explained in our previous study [14]. All sewersheds have independent sewer systems and wastewater treatment facilities. Daily confirmed COVID-19 case counts of the sewersheds were retrieved from the Ohio Coronavirus Wastewater Monitoring Network based on reported symptom onset time [27]. The sewersheds covered by this study are distinct in the scale of COVID-19 case incidences and SARS-CoV-2 viral loads in wastewater.

### Feature selection and engineering

Time and seasonality are important drivers of COVID-cases, which are the key components of various non-WBE based COVID-19 trend predicting models [22]. This study aimed to mine predicting information from the wastewater samples and the associated sewersheds to provide a more accurate prediction for the population in the community covered by the wastewater. Around 30 domain-specific features were extracted from wastewater samples and the community in the sewersheds. These features can be assigned to three categories: (1) viral loads (SARS-CoV-2 gene concentrations) and other biochemical parameters of wastewater (RNA, fecal indicators, and total suspended solid concentration, pH, temperature, etc.); (2) geographical parameters of the sewersheds (population, precipitation, etc.); and (3) and socioeconomic parameters of the communities (social vulnerability indexes: socioeconomic status, household composition & disability, minority status & language, and housing type& transportation as well as number of testing center) [28] (Fig 1). The socio-economic features were selected based on our previous study [29]. These parameters were determined to be significantly correlated with reported COVID-19 incidences and SARS-CoV-2 wastewater concentrations. To handle the skewness in the continuous features, normalization and Box-Cox transformation were conducted on all models except GBDT. Categorical features such as community name, sewer type (combined or separate), and weekend or weekday are one-hot coded.

It is expected that the SARS-CoV-2 gene concentration is one of the most powerful features, while other features may enhance the performance of the model in handling uncertainties in the wastewater data. Despite that non-time series models cannot learn from the short/long-term dependency of sequential data, wastewater surveillance features can be fed to the non-time series models for COVID-19 case prediction in the community represented by the sewershed. These features can also be uptaken by the time-series model like LSTM and Prophet to provide a more comprehensive wastewater monitoring of COVID-19 in the sewersheds.

### Model building and hyperparameter tuning

Data preprocessing was conducted using *scikit-learn* and *scipy*.*stats* package in Python 3. Models were further built with the Tensorflow framework (version 2.7.0). All models were trained and tested using a train/validate split ratio of 70% and 30%. A univariant linear regression model was built first to serve as a baseline using the SARS-CoV-2 viral loads only. Multivariate non-time series models were then trained for next-time-step COVID-19 case prediction in different communities. Data from all communities in different sewersheds are trained jointly. 5-fold cross-validation and grid search were performed for parameter tuning and model evaluation.

**Fig 2:**
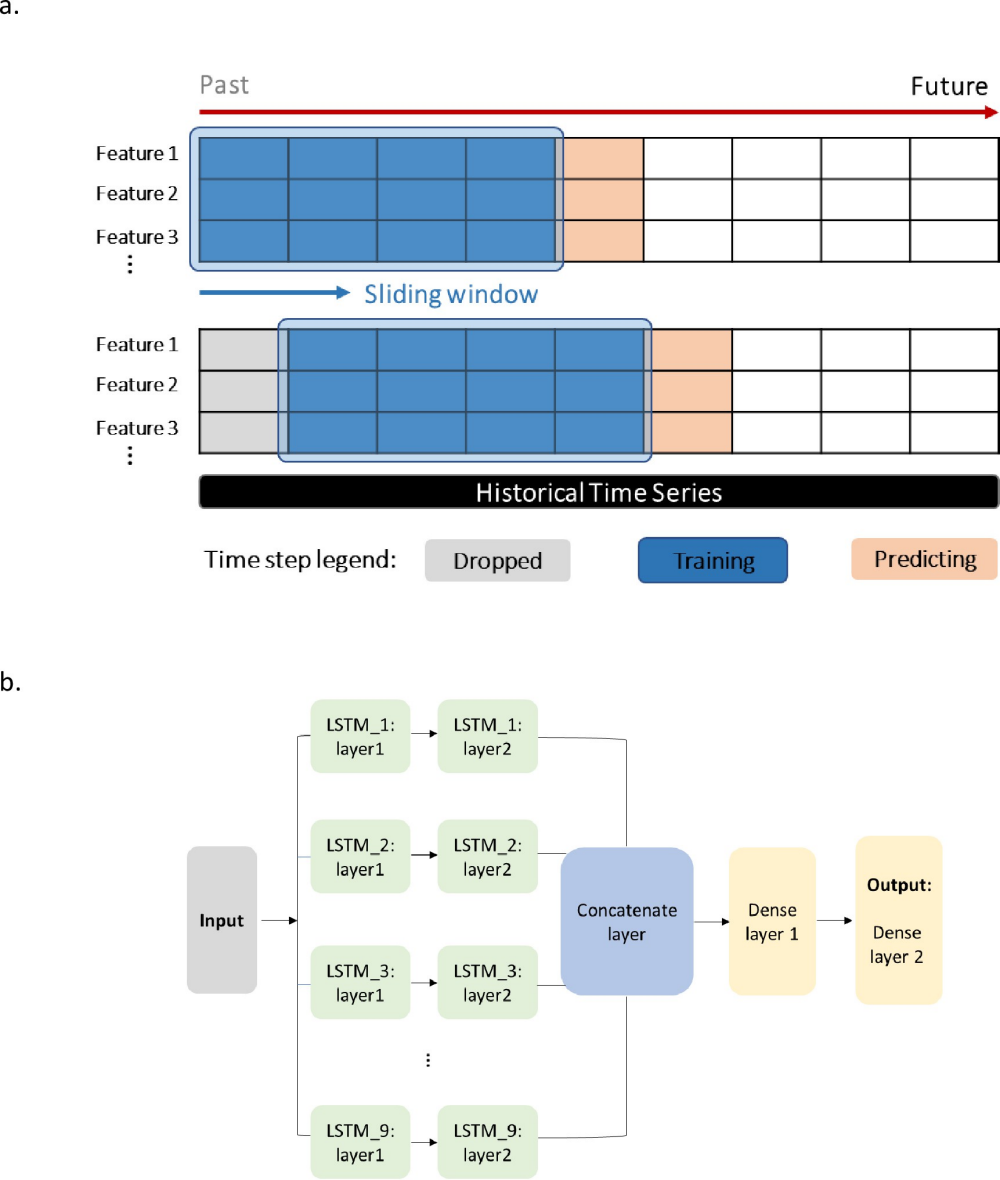
LSTM model flow. a) Input type and sliding window; b) LSTM model architecture.

To prevent data leakage, for time series models, the COVID-19 case in time step A is predicted from data from the past 16 time steps (sliding window of 8 weeks) in different communities (Fig 2A). Features are added as regressors to the Prophet model for training, and COVID-19 cases are predicted with a 95% credible interval. For multivariate LSTM, the architecture of the model is summarized in Fig 2B. For each paralleled time series from the 9 sewersheds, input multivariate feature matrixes were firstly taken by a stacked LSTM with two hidden layers (64 and 32 memory cells). Then the hidden states of the 9 LSTM layers were merged using a concatenate layer, followed by two dense layers of 32 and 9 units (output shape). The concatenating layer combined the features of each sewershed, which gave a higher level of feature abstraction and helped learn the dependency among the sewersheds to some extent. In this way, the WBE data from all 9 sewersheds were trained jointly, despite the fact that the LSTM layers were learned separately. A dropout rate of 0.05 and L1 & L2 regularizer value of 0.025 was adopted for the LSTM layers. Adam optimizer was employed to adapt the learning rate.

## Result and discussion

### Feature importance

**Fig 3:**
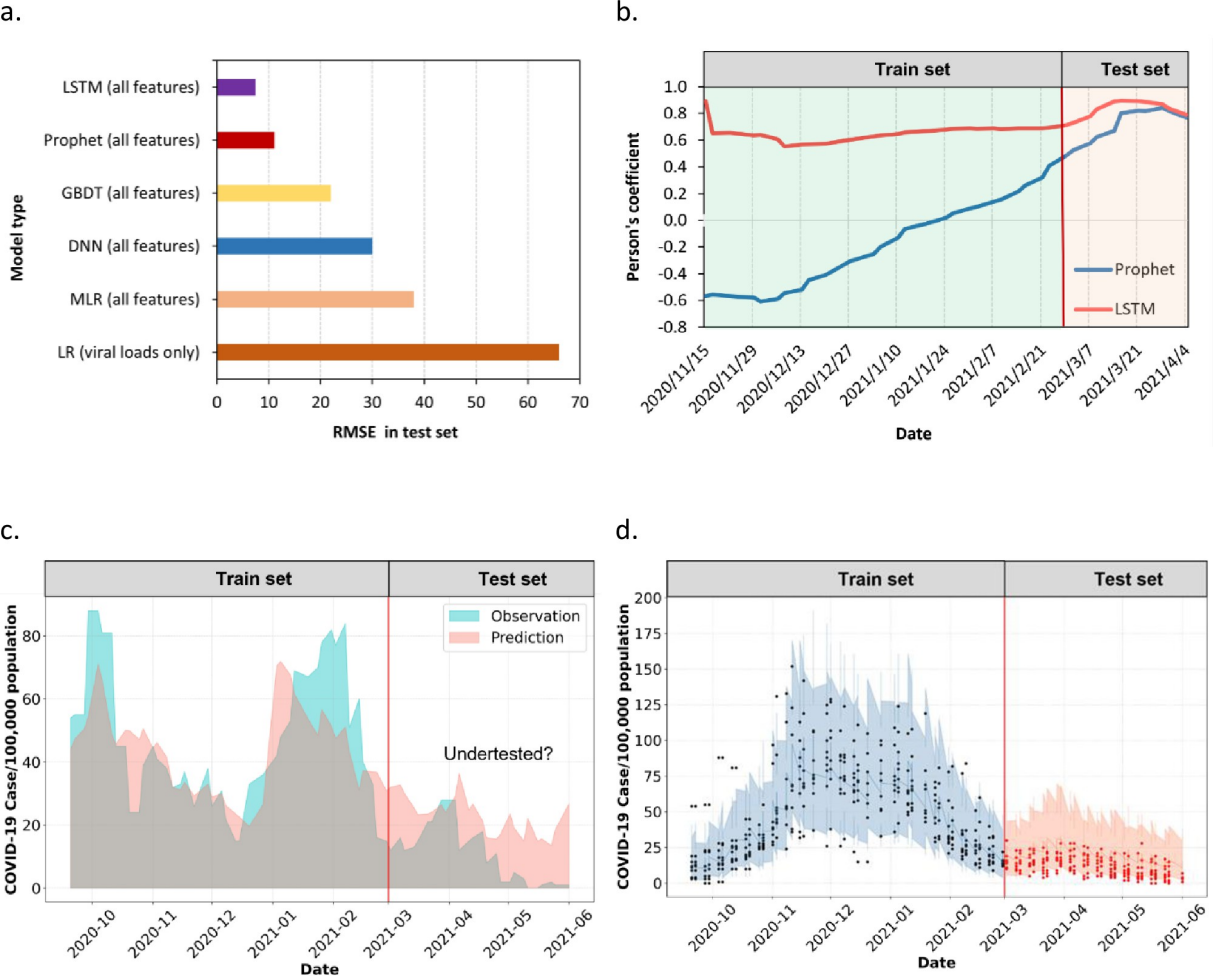
Comparison of model performance. a) RMSEs of five machine/deep learning models; b) Person’s correlation coefficient of the predicted (LSTM and Prophet model) vs. observed COVID-19 case numbers (15 days rolling average) for Athens (‘bad case’); c) LSTM model performance on the data from Athens sewershed in Ohio (Overlaid area plot of the predicted vs. observed COVID-19 case numbers. Potential undertesting was observed); and d) Prophet model on all sewersheds. The shaded area indicated the 95% credible interval of the model parameters. True observations are shown in solid dots.

The performance of the models was evaluated with the root mean square error (RMSE) metrics of the test set (Fig 3A). Compared to the baseline model (univariant linear regression model using the SARS-CoV-2 viral load feature only), the addition of other wastewater- and sewersheds-related features dramatically improved the accuracy of the MLR model. Permutation feature importance was measured to find the key features. Using the best performing model, LSTM, it is not surprising that SARS-CoV-2 viral loads, time, and sewershed identity are highly important. This finding supported the achievement of our overall goal, which is to build effective machine learning models to predict COVID-19 trends in multiple communities using the longitudinal data. Furthermore, although the significant contributing features slightly varied among different models, they covered all three feature categories. Among the wastewater parameters, total suspended solids, fecal indicator concentration, RNA concentration, flow rate, pH, and temperature are the key features besides viral loads. The accumulative number of COVID-19 testing sites, population, and precipitation is the main model contributors in the geographical parameters category. Within the socioeconomic parameters of the communities, social vulnerability indexes [28], and poverty are also important features.

### Model building and performance evaluation

Overall, time-series models outperform the non-time series models, with LSTM being the best. Prophet and LSTM are powerful models in jointly making long-term predictions for all communities/sewersheds. Compared to Prophet (training set R^2^ = 0.83, test set R^2^ = 0.63), LSTM model (training set R^2^ = 0.94, test set R^2^ = 0.81) derived higher R^2^s. Moreover, according to Pearson’s correlation coefficient between the predicted value and observed value, LSTM yielded higher predictive accuracy than Prophet for the Athens’ data, which is the ‘bad case’ among all sewersheds (Fig 3B). Our previous study about the correlation between the viral concentrations in wastewater and the daily COVID-19 confirmed cases identified as the only non-correlating sewershed among all 9 sites investigated (Ai et.al, 2021). Athens is a small college town with students as the major residence type. The confirmed case trend of Athens is different from other communities. Peak infection occurs in mid-September and early October during the student returning season, followed by a decline after that in November and December when the students were leaving campus for holidays. In addition, the discrepancy between the wastewater data and new case data can be observed, which might be explained by an underestimation of the cases as most students stay asymptomatic.

In other words, compared to Prophet, LSTM showed better generalizing capability among different communities, probably owing to the fully connected layer after concatenating the LSTM layers. This also indicates that the LSTM model is preferred when the communities are diverse in wastewater biochemical attributes, and geographical, and socioeconomic parameters, which is a typical case in real-world COVID-19 WBE projects. LSTM is known for its ability to handle high-dimensional data, while the capability of Prophet in that regard remained understudied. Owing to the bad case and the variations among the sewersheds, the performance of both models in the test set is not very satisfying. This performance can also result from underestimating the case number (Fig 3C), a relatively small dataset, and overfitting the model [18]. However, Prophet has its advantages over LSTM. As a Bayesian structural additive model, Prophet can provide seasonal trends and the credible interval of the prediction (Fig 3D) [25]. We also examined the potential lead time of WBE to the clinical case. In the LSTM model, the RMSE on the test set was improved by 10% after inducing a 5-day-lag time to the wastewater data, implying that the clinical reports can be backlogged.

Collectively, we recommend time-series machine learning models to deal with multi-site WBE data, especially LSTM. With proper feature selection, feature engineering, and hyperparameter tuning, we believe machine learning can be a powerful tool for predicting COVID-19 trends from wastewater surveillance.

### Limitations and future research perspectives

The feasibility of applying machine learning in the COVID-19 wastewater surveillance system is discussed in this article. However, further attention is needed in many other realms. First, WBE systems or networks on a larger scale are preferred due to the lack of standardization in wastewater sampling and processing methods. The resulting dataset can cover communities of different types. As mentioned above, one of the challenges for multi-community prediction is overfitting. For LSTM, we found L1 and L2 regularization and dropout to be effective methods. Removing redundant features and correct feature engineering can further reduce the risk of overfitting. As a pilot study, the dataset in this study is relatively small. Larger datasets can enhance the generalization of the models on various communities.

Due to the small dataset size, this study only focused on the next-time-point prediction with a sliding window of 16-time points. Future research is welcomed for developing WBE-based machine learning models to forecast the COVID-19 trends in a longer future time period. A longer or shorter sliding window is also worth examining. Other LSTM variants, such as bidirectional LSTM and encoder and decoder LSTM models, have been successfully applied to forecast the spread of COVID-19 in India [30]. Application of these models to WBE data might also be promising. It is also worth mentioning that in the temporal LSTM model of this study, the correlation between the different communities/sewersheds was only addressed by the dense layer after concatenation. This might be sufficient for distant communities with independent sewer systems and when the interaction between the COVID-19 dynamics in the communities is poor. Spatiotemporal models like convolutional neural network (CNN)-LSTM can be powerful in demonstrating the correlation between the communities when needed [31, 32].

Moreover, deployed models and wastewater testing approaches need to be updated constantly using upcoming WBE data. For example, the lead time of WBE results to clinical reports might vary among the communities and change over time. The reported COVID-19 case number can become less reliable due to the increased self-testing and vaccination rate [18]. When new variants emerge, the target genes in wastewater surveillance may need to be adjusted accordingly. Therefore, based on the model’s performance on the new data gathered, hyperparameter tuning needs to be conducted occasionally. A WBE-machine learning system can be built to help improve the efficiency of model deployment and updating. Weekly wastewater data can be uploaded by the researchers to an online platform. The data will be used to update the model via automatic parameter tuning tools like Keras-tuner under the supervision of a human expert, followed by automated data visualization [33]. This system can also be coupled with an alarming outbreak system. Decisions can be made based on whether the trend predicted indicates an outbreak or not, which can also be achieved by machine learning algorithms. Eventually, hotspots or areas of concern can be accurately identified.

## Conclusions

In this prolonged pandemic, wastewater surveillance is an effective complementary tool for COVID-19 monitoring. The development of predictive models is necessary but challenging due to the intrinsic complexity of the wastewater matrix and the variations in the sewersheds. Machine learning/deep learning are emerging techniques for WBE that have great potential to resolve the uncertainties in wastewater data. As the scale of wastewater surveillance is continuously expanding, it is crucial to adapt models for multi-community prediction. We proved that this adaption can be achieved with time-series deep learning models like LSTM and Prophet. The wastewater data and deployed machine learning models will need proper maintenance. To enable the automatic model deployment, updating, and interpretation, a machine learning system can be built in the future. Studies on machine learning-based long-term forecasting of COVID via WBE are also recommended.
